# Family caregivers’ perspectives on their interaction and relationship with people living with dementia in a nursing home: a qualitative study

**DOI:** 10.1186/s12877-022-02922-x

**Published:** 2022-03-16

**Authors:** Charlotte T. M. van Corven, Annemiek Bielderman, Peter L. B. J. Lucassen, Hilde Verbeek, Ivonne Lesman-Leegte, Marja F. I. A. Depla, Annerieke Stoop, Maud J. L. Graff, Debby L. Gerritsen

**Affiliations:** 1 Department of Primary and Community Care, Radboud Institute for Health Sciences, Radboud Alzheimer Center, Radboud University Medical Center, Nijmegen, the Netherlands; 2 Department of Primary and Community Care, Radboud Institute for Health Sciences, Radboud University Medical Center, Nijmegen, the Netherlands; 3 Department of Health Services Research, Living Lab in Ageing and Long-Term Care, Care and Public Health Research Institute, Maastricht University, Maastricht, the Netherlands; 4 Department of General Practice and Elderly Care Medicine, University of Groningen, University Medical Center Groningen, Groningen, the Netherlands; 5 Department of Medicine for Older People, Amsterdam Public Health Research Institute (APH), Amsterdam UMC, VU University Medical Center, Amsterdam, The Netherlands; 6 Department Tranzo, Scientific Center for Care and Welfare, Tilburg School of Social and Behavioral Sciences, Tilburg University, Tilburg, The Netherlands; 7 Scientific Institute for Quality of Healthcare and Department of Rehabilitation, Radboud Institute for Health Sciences, Radboud Alzheimer Center, Radboud University Medical Center, Nijmegen, the Netherlands

**Keywords:** Dementia, Family caregivers, Nursing homes, Social interaction, Psychosocial support

## Abstract

**Background:**

Social interactions are important for people living with dementia in a nursing home. However, not much is known about interactions and relationships between residents and family caregivers and related experiences of family caregivers. We aim to advance the knowledge on how family caregivers interact with people living with dementia in a nursing home and how they maintain or redesign a meaningful connection.

**Methods:**

Qualitative research using interviews with family caregivers (*n* = 31) to explore perspectives on their interaction and relationship with the person living with dementia. Interviews were held during the reopening of nursing homes after the first COVID-19 lockdown in the Netherlands. In this situation, family caregivers became more aware of their interaction and relationship with the resident, which provided a unique opportunity to reflect on this. The interviews explored the interaction and relationship in a broad sense, not specifically for the COVID-19 situation. Thematic analysis was performed to analyze the data.

**Results:**

We were able to identify three key themes reflecting the experiences of family caregivers: (1) changes in the interaction and relationship, (2) strategies to promote connection, and (3) appreciation of the interaction and relationship. From the viewpoint of family caregivers, the interaction and relationship are important for both the resident living with dementia and for themselves, and family caregivers have different strategies for establishing a meaningful connection. Nevertheless, some appear to experience difficulties with constructing such a connection with the resident.

**Conclusions:**

Our results provide a basis for supporting family caregivers in perceiving and establishing mutuality and reciprocity so that they can experience togetherness.

**Supplementary Information:**

The online version contains supplementary material available at 10.1186/s12877-022-02922-x.

## Background

For people living with dementia, interactions and relationships with their family and friends are especially important as these help them to fulfil their potential, live their lives with the highest possible degree of independence, and participate in social activities [1, 2]. The significance of these social interactions has been highlighted for people living with dementia in a nursing home as well [3, 4]. Specifically, relationships within families are imperative aspects of well-being in the everyday life of residents [5], and social interactions are associated with fewer neuropsychiatric symptoms [6] and positively affecting the person living with dementia [3]. It was also found that support from family promotes adjusting to life in the nursing home [7]. The recent COVID-19 pandemic highlighted the importance of social interactions, as meaningful connections came under pressure due to restrictive visiting policies [8, 9]. Furthermore, research on empowerment for people living with dementia, which includes a sense of identity, usefulness, control, and self-worth, showed that empowerment takes place within the interaction between the person living with dementia and their environment [10]. Loved ones of people living with dementia in a nursing home (called family caregivers in this article) could play an important role in this.

Nevertheless, not much is known about the interactions and relationships between people living with dementia in a nursing home and their family caregivers and related experiences of those family caregivers. They may experience challenges in connecting with the person living with dementia [11], as dementia causes loss of abilities across cognitive, functional, and behavioral domains. Preserving a sense of togetherness, for example feeling “one” as a couple, may be challenging [12, 13]. Previous research found that strong ties in the relationship are helpful in creating new ways of communication, for example, using body language when verbal communication becomes difficult [5], or developing strategies to preserve continuity in the relationship, such as scheduling visits for periods when residents are more alert and capable of interacting [13]. Nevertheless, such studies on the perspectives and experiences of family caregivers on interaction and relationships in the nursing home remain scarce, as research regarding family caregivers mostly focuses on caregiving for people living with dementia at home [14–16], or on family caregivers’ functional involvement in the nursing home, such as communicating with staff or making decisions on care and support [17, 18]. To support family caregivers in establishing a meaningful connection with the resident, more understanding on the perspectives and experiences of family caregivers appears valuable.

In this study, we aim to explore the perspectives of family caregivers on their interaction and relationship with the person living with dementia in a nursing home. We aim to advance the knowledge on how family caregivers interact with people living with dementia in a nursing home, their related experiences, and how they construct a meaningful connection.

## Methods

### Design

We performed a longitudinal qualitative study, interviewing family caregivers of people living with dementia residing in a nursing home in two stages. Data collection took place in the Netherlands from May 2020 to February 2021.

### Setting and participants

Family caregivers of residents living with dementia in a psychogeriatric unit from five nursing homes participated. Data collection was coordinated by the local university networks for long-term care in the regions of Amsterdam, Groningen, Maastricht, Nijmegen, and Tilburg. These local university networks are alliances between a university and multiple care organizations in the region. For each local university network, one nursing home participated in this study, each with 21 to 163 people living in psychogeriatric nursing home units. We interviewed a convenience sample of family caregivers in two stages. The consolidated criteria for reporting qualitative research (COREQ) were followed in this article [19], see Additional file 2.

#### Stage 1

Nursing homes in the Netherlands closed their doors to visitors on March 20^th^ 2020, as obliged by law, to prevent and control COVID-19 infections. This meant that family caregivers were unable to visit residents. After two months of lockdown, nursing homes in the Netherlands were cautiously reopened for visitors during a pilot, with strict guidelines, including one designated visitor being allowed per resident, and visits took place at least at 1.5 m distance [8, 9]. A convenience sample of family caregivers who participated in the pilot in one of the five participating nursing homes, and thus visited their loved one in May 2020, were asked by a contact person of the nursing home to participate in a telephone interview on their experience of the visit. All interviews took place in May 2020. It is not known how many participants were asked to participate in the interview.

#### Stage 2

The interviews from stage 1 provided interesting information on interactions and relationships between family caregivers and residents, but data saturation regarding overall experiences was not reached in this first stage, as the interviews focused on the impact of COVID-19 restrictions. Therefore, a second stage was added, in which a purposive sample of 20 participants were asked after four to nine months (September 2020 – February 2021) for a follow-up interview to collect more information on the overall interaction and relationship the resident. In approaching participants for the follow-up interview, we considered sex, relation to the resident, length of stay in residential care, and the region in the Netherlands to achieve variation in participant characteristics [20]. During these follow-up interviews, guidelines for visitations were still in effect, including one visitor at a time (from a few designated visitors per resident) and at least 1.5 m distance between the family caregiver and resident. However, not all dyads kept this 1,5 m distance.

### Data collection

To conduct the interviews in stage 1, the researchers (CvC, AB, DG) developed an interview guide, as shown in Additional file 1. Questions aimed to investigate general experiences with the first visit to the nursing home after it reopened for visitors after the first COVID-19 lockdown in the Netherlands, and were aimed at three topics: (1) organization of the visit, (2) impact on the family caregiver, and (3) impact on the resident living with dementia in the nursing home. General experiences regarding the visits are described in the article of Verbeek et al. (2020) [8]. As these interviews also revealed interesting information on interactions and relationships between family caregivers and residents in general, the interview guide was rigorously adapted (Additional file 1) for a second round of interviews (stage 2). Questions in these stage 2 interviews specifically aimed to investigate the overall interaction and relationship between family caregivers and people living with dementia in the nursing home and further explored information already given by family caregivers in the first round of interviews. Data collection continued until saturation was reached. The focus of this article is the overall interaction and relationship between family caregivers and residents living with dementia of nursing homes.

The researcher of the local university network for long-term care called the participant to make an appointment for the interview. In all interviews, open-ended questions were asked, followed by questions on themes that were introduced by participants. No pilot was done before data collection took place. Semi-structured interviews were done via telephone and lasted between 12 and 68 min for stage 1, or between 16 and 38 min for stage 2. Interviews were performed by seven interviewers (CvC, AB, IL, EdV, EvV, MJ, CB) in stage 1, and by the first author (CvC) in stage 2. All interviewers were female and had experience with conducting interviews. Before starting the interview, interviewers introduced themselves and explained the aim and reason for the study. Participants were given the opportunity to ask questions, and provided oral consent. Interviews were tape-recorded and transcribed verbatim. Transcripts were not returned to participants for comments or correction. Field notes were written after each interview.

### Data analysis

Firstly, transcripts of the interviews from stage 1 regarding visiting guidelines were entered into Atlas.ti (version 8.4.15). Thematic analysis was used [21], in which common themes and categories were identified using inductive reasoning and constant comparison, which means that no theoretical perspective guided the coding or interpretation. We developed a coding system by using open codes to describe all relevant aspects raised by participants [22]. Thematic analysis was also used for the follow-up interviews. As open codes were used to describe all new relevant aspects raised by participants, the coding system was rigorously adapted for follow-up interviews in stage 2. Coding of the interview transcripts from stages 1 and 2 was done separately by two researchers (CvC, MW). Codes referring to the same phenomenon were grouped into categories, and these categories were grouped into higher-order themes. Consensus meetings with the research team were held to reach agreement on coding and interpretation, and categories and themes were defined together.

## Results

### Participant characteristics

In total, 31 family caregivers participated in this qualitative study – 30 family caregivers participated in an interview in stage 1, and 13 participated in a follow-up interview in stage 2. Twenty caregivers were asked to participate in the follow-up interview (considering variation in participant characteristics), six of whom did not participate: three did not respond, two did not provide a reason for not wanting to participate, and one did not participate since the resident had died since the first interview. One family caregiver only participated in the follow-up interview. Characteristics of the participants are shown in Table 1. For two family caregivers participating in both interviews, both parents resided in the nursing home – either both or one parent living in a psychogeriatric nursing home unit. For two family caregivers, the resident had died at the time of the follow-up interview. Interviewers did not have any relationship with participating family caregivers prior to study commencement.

**Table 1:** Characteristics of participating family caregivers

	**All participating family caregivers** (*n* = 31)	**Family caregivers participating in follow-up interview** (*n* = 14)
	Mean (SD) or n (%)	Mean (SD) or n (%)
Mean age (years)	63.3 (9.9), range 48 – 84^b^	65.7 (7.9), range 56 -78^b^
Sex (% female)	24 (80.0)	11 (78.6)
Relationship to resident		
Married / partner	5 (16.7)	3 (21.4)
Child (in-law)	19 (63.3)	8 (57.1)
Brother or sister	1 (3.3)	1 (7.1)
Niece or nephew	4 (13.3)	1 (7.1)
Other	1 (3.3)	1 (7.1)
Travel time to nursing home (minutes)	17.0 (16.4), range 0 – 75^b^	15.4 (14.2), range 5—60
Average number of visits^a^		
(Almost) every day	6 (20)	2 (14.2)
A few times per week	16 (53.3)	10 (71.6)
Once per week	5 (16.7)	1 (7.1)
Less than once per week	2 (6.7)	0 (0.0)
Average visiting time^a^		
Approximately half an hour	0 (0.0)	0 (0.0)
Approximately an hour	9 (30)	4 (28.6)
More than one hour	17 (56.7)	9 (64.3)
Length of stay of resident in nursing home (years)	3.1 (3.0), range 0.5 – 15^b^	2.8 (1.6), range 1—6

^a^Before the start of the COVID-19 pandemic

^b^One missing for age, average number of visits and number of years living in nursing home, four missing for average time of visit for all family caregivers, one participating in the follow-up interview

### Findings of the interviews

Based on the perspectives of family caregivers, we were able to identify three themes in the interaction and relationship between people with dementia living in a nursing home and their family caregivers: (1) changes in the interaction and relationship, (2) strategies to promote a connection, and (3) appreciation of the interaction and relationship. Table 2 shows an overview of the codes, categories and themes. Saturation was reached; none of the categories or themes emerged after analysis of the second follow-up interview, and after the twelfth follow-up interview no new relevant codes emerged.

**Table 2:** Overview of themes, categories and codes

Themes (heading) and subthemes	Codes
**Changes in the interaction and relationship**	
Changes in communicative abilities of the resident	*Resident shows no initiative, cognitive abilities vary*
Quality of the changed interaction and relationship	*Relationship has improved, making a connection is not possible*
Experiences with the changes in interaction and relationship	*Visit feels as long, difficult to lose connection*
**Strategies to promote connection**	
Verbal interaction	*Chatting together, family caregivers learn suitable attitudes over time*
Undertaking activities	*Going outside together, preserving interests of resident*
Physical interaction	*Physical interaction is essential in interaction, caregiving promotes intimacy*
Just being there	*Visiting so residents ‘feel’ their presence, nursing home feels as home for family caregiver*
Contextual strategies	*Visiting at ‘the right time’ of the day*
**Appreciation of the interaction and relationship**	
Appreciation by residents living with dementia	*Family caregivers cannot be replaced by healthcare professionals, resident smiles when seeing the family caregiver*
Appreciation by family caregivers	*Supporting during the last life phase is fulfilling, family caregiver is happy when the resident is happy*

### Theme “Changes in the interaction and relationship”

One of the key themes that emerged from the analysis regarded changes that occurred in the interaction and relationship between the resident and the family caregiver. This included changing communicative abilities of the resident, the subsequent influence on the quality of the interaction and relationship, and pertaining experiences of these changes by family caregivers.

### Changes in communicative abilities

Family caregivers reported that the resident’s dementia changed their interaction and relationship. They stated that the resident’s communicative abilities decreased, mostly describing the decrease inverbal interaction. Examples ranged from decreased cognitive abilities limiting in-depth conversations to residents who did not respond to family caregivers or showed no signs of recognition. Many family caregivers mentioned that the residents’ initiative in interaction disappeared and that conversations (if there were any) were more and more about the past:

*My mother doesn’t say much. If you ask my mother: how are you doing? Then she just smiles and nods, but says nothing. […] She is just very withdrawn at the moment, very much in her own little world. […] That is probably the deterioration in her condition. But she does recognize me, and she knowns exactly who I am, but she just doesn’t have anything to say anymore. (daughter, P43)*

Interviewees reported that the cognitive abilities of the resident declined but that these abilities could fluctuate from day to day. They stated that on some days the resident was more in their own world, confused, “far away”, or showed few emotions. A daughter explained that due to this fluctuation, the interaction with her father differs between visits:

*If he is in that fantasy world, well, then he tells me that he went shopping everywhere by car. But yeah, I know that is not true at all. He is caught up in the story and the experience. I just let him be. I don’t tell him: that’s not right. Then I ask more questions, but these are, of course, pointless conversations, because I actually know that these things didn't happen at all. If he is having a good day, then you can also talk with him about my nephew or about my partner or about our house we built. If he Is having a good day he knows all of this again. (daughter, P26)*

Barriers for interaction that were mentioned included sadness in the resident, anger, tiredness, or moments of distraction. Furthermore, family caregivers mentioned barriers to interaction such as physical discomfort, drowsiness due to medication use, or limited hearing.

### Quality of the changed interaction and relationship

Although all respondents stated that the resident’s communicative abilities had changed, their experiences with the quality of their interaction and relationship with the resident differed considerably. Many family caregivers stated they generally succeed in connecting with the resident. Several interviewees stated that good interaction with the resident is self-evident, as there had always been a strong relationship. As the partner of a resident stated:

*Interviewer: Did you have the feeling you could make a connection as usual with your husband?*

*Family caregiver: Yes, but that makes sense, we’ll be married 55 years next week. (wife, P01)*

Some family caregivers mentioned that the interaction and relationship with the resident had improved as dementia progressed. A son explained:

*My father was always a very independent man. Actually, the contact with my father was never very intimate, but towards the end he surrendered completely to me. I arranged everything for him. I just noticed that he was glad when I was there, and that he also kind of put the responsibility entirely with me. (son, P09)*

Others stated that the quality of the interaction and relationship with the resident decreased as the dementia progressed. Several family caregivers reported the interaction had become more superficial over time. Others mentioned they failed to connect with the resident.

*Making a connection is very difficult anyway. You can’t go through everyday things with her anymore, because it is all just too much for her. (husband, P32)*

### Experiences with the changes in interaction and relationship

Many interviewees reported experiencing the changes in interaction as difficult. They reported struggling when seeing cognitive abilities decrease, or with the feeling of losing connection with the resident.

*That is difficult, if you notice that the connection is getting worse. And the idea that he will no longer recognize you, that is difficult. (brother, P40)*

Many family caregivers reported challenges when visiting the resident, although related experiences were different. Some family caregivers stated *it takes a certain type of character* to connect with people living with dementia, which some say comes naturally to them.

*I think you have to be a certain type of person for that, and I am. Even if I don’t get an answer, I still sit with him and grab his hand. I talk to him, and so he will hear me talk. And then, yes, he gives a reaction. I try to do it that way. (daughter, P46)*

Other family caregivers explained that a visit to the nursing home sometimes feels quite long. They reported that having a conversation was challenging and stated preferring to have some distractions during the visit. They mentioned examples such as having the television playing, undertaking activities, or having other people to talk to, such as other family or friends, other residents, or healthcare professionals. This view was not shared by all participants, as some family caregivers mentioned that a connection was established best when they were alone with the resident.

*If my sister or my brother is there, and we go for a walk, then it breaks the visit so to speak. It’s a little hard when she doesn’t talk. Well, sometimes a healthcare professional walks in, and then you usually have a chat. Or someone from the cleaning service, who also chats a bit. That breaks the visit for a short moment. (daughter, P34)*

*Because she, of course, says the same thing a hundred times, and at some point, I don’t really know what to say anymore. (friend, P16)*

Some family caregivers mentioned having accepted the changes in the interaction or relationship or having got used to them.

*We do have a conversation, but he often just replies yes or no, you know. […] That’s not nice, but, of course, I’ve accepted that for a while now. I’ll live with that. So yeah, as long as I’m there, you know, that he feels that I am there. (daughter, P46)*

*If I know he is feeling good at that moment, […] then I actually feel just as good. I think it is also a bit of resignation, like, this is how it is and now we should try to be happy. (wife, P01)*

A daughter explained she had accepted her father’s dementia with the accompanying changes in interaction. Nevertheless, situations in which her father talked about her mother as if she was still alive kept feeling confrontational to her.

*When he makes up those stories that he’s been out and about, I find it very easy to say: oh yeah and was it fun? I don’t have a problem with that. But when it comes to my mother, it feels much more complicated. […] For me, my mother has died of course, and for him she hasn’t. (daughter, P26)*

### Theme “Strategies to promote connection”.

The second key theme that emerged from the analysis comprised strategies family caregivers use to connect with the resident. These included verbal interaction, undertaking activities, physical interaction, ‘just being there’, and contextual strategies.

### Verbal interaction

With regard to verbal interaction, some family caregivers mentioned having learned the right approach to connect with the resident over time, for example, not asking difficult questions or not correcting the resident. Family caregivers also noted that using humor may help to distract from dreariness and that laughing together brings joy.

Several family caregivers mentioned that verbal interaction with the resident improved if other people were present. For example, a daughter mentioned that if a healthcare professional joined the conversation, she could also interact with the healthcare professional, which was favorable for her mother, as she heard the chitchat and felt she was part of the conversation. On the other hand, one family caregiver emphasized the importance of involving her mother in the interaction when her sisters were also present. She named the pitfall that the sisters only talked to each other while their mother was not involved in the conversation.

### Undertaking activities

Many family caregivers reported undertaking activities to connect with the resident. They mentioned activities including going outside, going for a walk, drinking coffee, and reminiscing, for example, by looking at pictures. Moreover, family caregivers mentioned connecting activities such as singing, listening to music, watching television, but also activities such as re-organizing the wardrobe together. Some family caregivers mentioned trying to undertake activities to connect to the person someone always used to be.

*Then you just see that she is happy, when I do that, do a bit of laundry with her. Because that used to be her thing. So, I just involve her. (friend, P16)*

Furthermore, a family caregiver mentioned that the caregiving task of helping with eating was a way for her to interact with the resident.

*Lately, I often visited around dinner time, so I could help her eat, or at least offer her food. I thought that was a nice thing to do, because you have some kind of connection. […] If you helped her eat, you could say something like: here’s another bite. Then she would open her mouth and sometimes she would say: nice. Or that I saw that she was thirsty and I asked: do you want some more water? So, there was still some form of communication possible. […] Helping her eat has really become a form of communication for me. (daughter, P18)*

Family caregivers noted that they distracted the resident from depressed moods by undertaking activities.

*When I arrive at my mother’s room, she is often quite depressed, but you can easily distract her by taking her downstairs for a while, drinking a cup of coffee, and then she will have forgotten all about it. (daughter, P44)*

Interviewees mentioned that possibilities for activities had decreased over time due to their own decreased physical health (e.g., not being able to push a wheelchair anymore), or the resident’s decreased cognitive abilities or physical health.

### Physical interaction

Many family caregivers reported on the importance of physical interaction for connecting with the resident, and to have missed this when it was not allowed due to COVID-19 restrictions. Although this importance differed between family caregivers, many family caregivers considered physical interaction essential. Some family caregivers mentioned that they increased physical interaction as dementia progressed, thus replacing verbal interaction. Interviewees also mentioned that residents appreciate physical contact, and it can fill silences.

*Oh, she was so happy. Yes, she was so happy. Touching me all the time. And she said: oh I haven’t touched you for so long. Yes, that’s really the most important thing for her. (daughter P02)*

*At times when the conversation normally stops for a while, then you stroke his head, or … Then you actually fill it up with physical contact and a little hug. (daughter, P22)*

Some family caregivers stated that physical interaction during caregiving tasks may increase feelings of connection to the resident.

*If we go to a restaurant and she has to go the bathroom, I help to get down her stockings. We are then in that toilet together. I don’t mean that it is nice to be in the toilet with someone, but you know what I mean, then you just have some kind of intimate connection. (niece, P45)*

On the other hand, other family caregivers stated that physical interaction is not important in their relationship with the resident. Mostly, these family caregivers mentioned that physical interaction had never been important, even before dementia onset.

### ‘Just being there’

Some family caregivers mentioned connecting to the resident by ‘just being there’. As a son said about the interaction with his parents:

*There are also people, for example, who don’t like silences if they are visiting somewhere. I don’t feel that way at all. I can sit down with my parents and say nothing for half an hour, and then there is still a feeling of connection. […] That’s just the warmth you give each other. (son, P09)*

These family caregivers reported being part of the daily routine of the resident, and feeling at home in the nursing home. As the partner of a resident mentioned:

*When I do the laundry, he usually comes to watch and help. And otherwise he just sits comfortably in the chair. He has a relaxing chair, and he just sleeps for a bit. And I’ll just do my thing. […] It’s feels a bit like a home life. (wife, P01)*

### Contextual strategies

Family caregivers mentioned that contextual strategies promoted interaction with the resident, including the strategy to visit during “the best time of the day of the resident”, and to visit at a place with which the resident is familiar.

*She’s often more confused after 3 pm, so I prefer to go early in the afternoon. (daughter, P02)*

### Theme “Appreciation of the interaction and relationship”

The third key theme that was identified is the appreciation of the interaction and relationship by people living with dementia and family caregivers.

### Appreciation of the interaction and relationship for residents living with dementia

Family caregivers reported that their visits mostly had a positive impact on the resident. They stated that they saw the resident enjoying their visit, feeling at ease, their mood improving, looking happy, or smiling. One family caregiver also mentioned that her dad said he enjoyed her visit.

*He does mention that. He has some very good moments, and then he says: oh I’m so glad you’re here. (daughter, P26)*

Furthermore, a family caregiver mentioned that the resident liked her visits, because they break up his day. Another interviewee mentioned that the resident liked that she brings him in contact with the world outside of the nursing home. Also, one family caregiver mentioned that her husband was more willing to accept care when she is around. Moreover, family caregivers reported that the resident apparently liked their visits, since the resident became grumpy when they leave, or asked healthcare professionals many times for them. One family caregiver stated that for these benefits of visiting, it did not matter who was visiting, but it had to be someone familiar for her dad.

*If it is me or my sister, you know, that won’t make much difference. But in any case, there’s someone whose name he recognizes and who is here. (daughter, P18)*

Multiple interviewees mentioned that their visits support the resident’s cognitive or functional abilities, and that these abilities would decline if they did not visit the resident. They did this by undertaking extra activities with the resident, or talking to them while incorporating their life history into the conversations, which healthcare professionals cannot.

*Talking to my mother is mainly based on memories, you see. How things used to be, bringing things up: do you remember this and this? And healthcare professionals cannot do that, of course. That’s why it seems she is just nodding off a bit. (son, P09)*

Some family caregivers also mentioned that they see the resident enjoying the visit, but stated they think that the resident would not notice if they would stop visiting. Other family caregivers added they questioned whether the visit sticks with the resident.

*She was very happy. But who visited on Mondays, for her that’s of course… So yeah, she was very happy and she really enjoyed it […], but I don’t know if it actually sticks. (daughter, P35)*

On the other hand, several interviewees mentioned that their visit could be tiring for the resident, or they wondered whether the resident always appreciated the visit.

### Appreciation of the interaction and relationship for family caregivers

Family caregivers reported that the interaction and relationship with the resident gave them satisfaction, as they supported their last life phase and having a good farewell. Nevertheless, they stated this was not always easy.

*For me it’s a form of saying goodbye in a good way. Suppose she doesn’t wake up at some point or a different situation arises, then I’ll still have the feeling that I did everything, I gave her what she longed for. (son, P09)*

*It may not be a nice time for him, I am very aware of that. Because he sometimes makes it clear that he doesn’t want to live anymore. When he is not feeling well, or is in pain, or nothing is going his way. Then it’s not fun, not even for me, because then I think: yeah, I can’t do anything about it, I can only try to make things nice and try to give attention and also ask the healthcare professionals if they want to do that. You know, that he can make his way through life a little. (daughter, P46)*

Moreover, family caregivers mentioned interaction with the resident gave them energy, or they enjoyed hearing stories about the past. Some family caregivers mentioned they enjoyed the interaction most when they saw the resident was thankful for their presence.

*My father is sometimes very grateful. He then takes me by the shoulders and rubs my shoulders. He smiles at me, and I just know that he is very happy and that gives me so much energy. Then I just want to put him in a little box and take him home (daughter, P29)*

Interviewees mentioned being grateful when the resident made a compliment, showed appreciation, or recognized the family caregiver.

*Because if he says to you, well, that you look nice, when I was wearing a new blouse... So, something like that, I like, yes. That he says that. That he notices that I’m wearing something new, or something like that. (wife, P30)*

Some family caregivers stated it was important for them to take care of practical matters, such as finances, the laundry or to monitor physical health. Interviewees reported that COVID-19 visiting policies restricted this.

*I am very happy that we can visit. But I’d really like to do something too. Id’ like to clean up some stuff in my dad’s closet or whatever. […] Staff don’t have the time for that, and I think: I would like to do that. (daughter, P29)*

Moreover, family caregivers reported they continued to visit and benefited from the visit, even though they found it difficult to interact, since “*it is your father*” or “*you just do it*”. As family caregivers said:

*Interviewer: What does the interaction with your father bring you?*

*Family caregiver: That’s a difficult question. I think it mainly has to do with him just being my father, and of course he has done a lot for me in my life. That I also want to give him something back. But in terms of the conversation, it is a rarity that you can really discuss something of added value with him. Those occasions are exceptions. (daughter, P26)*

## Discussion

Three themes were found that reflected perspectives of family caregivers on their interaction and relationship with their loved one living with dementia in a nursing home: (1) changes in the interaction and relationship, (2) strategies to promote connection, and (3) appreciation of the interaction and relationship. Our results show that some family caregivers experience difficulties in making a meaningful connection with the resident, while others succeed in constructing togetherness despite decreased communicative abilities of the person living with dementia, for example, by undertaking activities or by *just being there*. Nevertheless, all family caregivers experienced benefits of their interaction and relationship with their loved ones in the nursing home.

This study confirms the importance of a meaningful connection between people living with dementia in the nursing home and their family caregivers, as family caregivers reported benefits for both. For people living with dementia, this included enjoyment and improved mood, and for family caregivers, this included feelings of satisfaction and fulfillment. Nevertheless, we found great diversity in the day-to-day visiting experiences of family caregivers. For some, establishing or maintaining a meaningful connection with the resident came naturally, while others experienced difficulties, such as decreasing mutuality and reciprocity in the relationship. A previous study among family caregivers of people living with dementia in the community has shown that perceiving mutuality by family caregivers requires them to direct additional attention to subtle positive responses from the person living with dementia [14]. Family caregivers in the nursing home may also benefit from acknowledging such responses as conveying affection or appreciation. It appears valuable for future research to explore how to support family caregivers in perceiving mutuality and reciprocity.

Furthermore, family caregivers applied different strategies to construct a meaningful connection with their loved one living with dementia, including verbal interaction, undertaking activities, physical interaction, ‘just being there’, and contextual strategies. These results are in line with previous research. For example, a recent study found that physical proximity and peaceful silence helps to reach emotional connectedness with the person living with dementia [23]. Nevertheless, the identified strategies for constructing togetherness do not appear to differ between family caregivers who succeed and those who experience difficulties in connecting with the resident. For example, going for a walk may help in interacting and connecting for one family caregiver, but may be a way to avoid interaction for others. The used strategies, therefore, do not guarantee a meaningful connection but are a useful starting point for supporting family caregivers in establishing a meaningful interaction and relationship, and so promote positive experiences [24, 25]. Strategies need to be tailored to the needs and wishes of people living with dementia and their family caregivers, and to their personal context.

Moreover, supporting a meaningful connection is expected to not only benefit family caregivers but also be beneficial for people living with dementia, as meaningful interaction can be empowering. Meaningful interaction with family caregivers may promote a sense of identity, usefulness, control, and self-worth [10], which are central to empowerment. It requires family caregivers to be aware of their role and corresponding attitude in the empowerment process [26, 27]. An empowering approach encourages the person living with dementia to be a person with individual talents and capabilities and may contribute to reciprocity in relationships [28]. The results of our study provide more details on barriers family caregivers experience and strategies they apply in establishing meaningful interaction. It provides a basis for supporting family caregivers to promote empowerment for people living with dementia in a nursing home.

It is interesting to note that in the interviews with family caregivers on the COVID-19 visiting policies, family caregivers highlighted the importance of not being able to act as caregiver, including household activities or checking finances, whereas in follow-up interviews asking specifically what made their interaction and relationship with the person living with dementia meaningful, this role of caregiving was rarely mentioned. Previous research showed that caregivers indeed wanted to continue having an active role in caring after nursing home admission [29]. They gained from the caring itself, including satisfaction, emotional reward, and personal growth, but also from the interaction between the family caregiver and the person living with dementia, including relationship gains and satisfaction in reciprocity [15]. Future research should be undertaken to explore the possibly stimulating role of caregiving for family caregivers of people living with dementia in a nursing home, as this may help family caregivers being meaningfully included in the nursing home life of their loved one [30–32], and being perceptive to the positive aspects of their interaction and relationship with the person living with dementia. These positive caregiving experiences can increase caregivers’ well-being [33].

### Strengths and limitations

To our knowledge, this is the first study to explore the perspectives of family caregivers on their interaction and relationship with their loved ones living with dementia in a nursing home. A key strength of this study is the timing of the interviews, as the lockdown and subsequent visiting guidelines facilitated family caregivers in reflecting on their interaction with the resident and their pertaining visiting routines, since these guidelines had hindered their usual ways of face-to-face interaction. Family caregivers appeared to become more aware of what made their interaction with the resident meaningful [8, 9]. Another strength of this study is the investigator triangulation, as multiple researchers were involved in conducting the first-stage-interviews [34]. All interviewers worked at a university network for long-term care, and therefore had experience and knowledge regarding the topic of our study. The relevance of our topic was confirmed as in the interviews of every interviewer interesting information came up about the interaction and relationships between family caregivers and residents. Also, were multiple researchers involved all analyses, having regular discussions with each other, so reaching agreement on the different themes. Last, all coding was conducted separately by two researchers, who had regular discussions with each other and the research team, and many discussions about the analyses were held with the research team, which increases the trustworthiness of the results [35].

A limitation of this study is the potential selection bias towards involved family caregivers visiting the nursing home often, as participants were a convenience sample from family caregivers who visited soon after the reopening of nursing homes after the first COVID-19 lockdown. Furthermore, given their heterogeneity, we think our study population reflects a wide range of family caregivers. A second limitation may be that some themes may have been overemphasized, such as the importance of a physical connection, because of the COVID-19 situation at the time of interviewing.

## Conclusion

Based on the perspectives of family caregivers, we conclude that the interaction and relationship between family caregivers and their loved ones living with dementia in a nursing home are important for both and that family caregivers apply different strategies for constructing togetherness. Nevertheless, some family caregivers appear to experience difficulties in establishing a meaningful connection with the resident. Our results provide a basis for tailoring interventions aimed at supporting family caregivers in perceiving mutuality and reciprocity in the interaction and relationship with the resident, by supporting family caregivers to understand and come to terms with changes that threaten the maintenance or establishment of a meaningful connection. This may help them to have a positive attitude, so maintaining or improving the quality of the relationship between family caregivers and residents living with dementia.

## Supplementary Information

**Additional file 1.** Topic guides.

**Additional file 2.** Consolidated criteria for reporting qualitative research (COREQ).

## Data Availability

The datasets generated and analysed during the current study are not publicly available to ensure participants’ privacy, but are available from the corresponding author on reasonable request.
