# Supplementary material for: Family caregivers’ perspectives on their interaction and relationship with people living with dementia in a nursing home: a qualitative study
Source: BMC Geriatr. 2022 Mar 16;22:212. doi: 10.1186/s12877-022-02922-x (PMC8924349; doi:10.1186/s12877-022-02922-x)
Supplement: Supplementary file 1 — Additional file 1. Topic guides. [file 12877_2022_2922_MOESM1_ESM.docx]

# Additional file 1: topic guides

## Topic guide first interview

**1. Organization**

**Planning the visit**

- How did you experience the information prior to the visit? How did you know visitors were allowed back into the nursing home?
- To which extent were you able to decide when you wanted to visit?
- Did you know what to expect during the visit?
- Did you know what was expected of you during the visit? For example, wearing a protective face covering.

**Allowing visitors back into the nursing home**

- How do you feel about being allowed back into the nursing home as visitor?
- To what extent was it difficult to decide who was allowed to visit your loved one?
- What do you think of this way of visiting?

**Visiting guidelines**

- What did you think of the guidelines for the visit?
- How was your contact with healthcare professionals during the visit?
- Was it difficult for you to comply with the guidelines? If yes, in which way?
- Did the guidelines affect the contact with the resident in the nursing home? If yes, in which way?
- Did you experience any barriers due to the guidelines? If yes, which barriers?

**Points for improvement**

- Is there anything you would like to be different on your next visit?

**2. Impact on the family caregiver**

- How did you experience the visit?
- Did you have any doubts about visiting? If yes, about what?
- Did the visit meet your expectations? Why?
- Was it of added value to see the resident of the nursing home in person? If yes, in which way?
- What made this visit different than visits before the COVID-19 pandemic?
- How did you keep in touch with the resident in the nursing home when visitors were not allowed? How did this visit differ from those alternative ways?

**3. Impact on the well-being of the resident living with dementia**

- Were you able to interact meaningfully with the resident? If yes, how did you meaningfully interact? What this different from visits before the COVID-19 pandemic? If yes, why?
- According to you, did the resident realize you had not seen each other for a long time?
- Did the resident seem different to you than during visits before the COVID-19 pandemic?
- According to you, how did the resident experience the visit?
- Was the resident sad during the visit? If yes, how did you notice?
- Was the resident happy during the visit? If yes, how did you notice*?*
- Was the resident anxious during the visit? If yes, how did you notice*?*
- Was the resident confused during the visit? If yes, how did you notice*?*
- Did the resident laugh during the visit?
- Was the resident enthusiastic during the visit? If yes, how did you notice*?*
- Were these emotions different than during visits before the COVID-19 pandemic?
- Did the resident feel at ease? If yes, how did you notice?

## Topic guide follow-up interview

**Meaningful interaction and relationship**

- How would you describe the relationship with the resident?
- What does a typical visit generally look like?
- In what way do you meaningfully interact with the resident?
- What makes you feel that you have meaningfully interacted with the resident?
- According to you, how does the resident experience your visits? How do you notice?
- In which situations do you succeed in interacting meaningfully?
- In which situations do you not succeed in interacting meaningfully? How do you cope when this happens?

**Presenting the results of the first interviews**

Participants mentioned that meaningful interaction can be established by (1) verbal interaction, (2) physical interaction, and (3) undertaking activities together.

- Are there other ways in which you meaningfully interact? According to you, what is the most important for meaningfully interaction?
- To what extent has meaningful interaction changed since the resident was diagnosed with dementia or moved to a nursing home?
