# Supplementary material for: Family caregivers’ perspectives on their interaction and relationship with people living with dementia in a nursing home: a qualitative study
Source: BMC Geriatr. 2022 Mar 16;22:212. doi: 10.1186/s12877-022-02922-x (PMC8924349; doi:10.1186/s12877-022-02922-x)
Supplement: Supplementary file 2 — Additional file 2. Consolidated criteria for reporting qualitative research (COREQ). [file 12877_2022_2922_MOESM2_ESM.docx]

# Additional file 2: consolidated criteria for reporting qualitative research (COREQ)

| Topic | Guide questions / description | Reported on page number |
| --- | --- | --- |
| Domain 1: research team and reflexivity | | |
| Personal characteristics | | |
| 1. Interviewer/facilitator | Which author/s conducted the interview or focus group? | 6 |
| 2. Credentials | What were the researcher’s credentials? *E.g. PhD, MD* | 1 |
| 3. Occupation | What was their occupation at the time of the study? | 24 |
| 4. Gender | Was the researcher male or female? | 6 |
| 5. Experience and training | What experience or training did the researcher have? | 6 |
| Relationship with participants | | |
| 6. Relationship established | Was a relationship established prior to study commencement? | 7 |
| 7. Participant knowledge of the interviewer | What did the participant know about the researcher? *E.g. personal goals, reasons for doing the research* | 6 |
| 8. Interviewer characteristics | What characteristics were reported about the interviewer/facilitator*? E.g. bias, assumptions, research and interests in the research topic* | 6 |
| Domain 2: study design | | |
| Theoretical framework | | |
| 9. Methodological orientation and theory | What methodological orientation was stated to underpin the study? *E.g. grounded theory, discourse analysis, ethnography, phenomenology, content analysis* | 6 |
| Participant selection | | |
| 10. Sampling | How were participants selected? *E.g. purposive, convenience, consecutive, snowball* | 4 |
| 11. Method of approach | How were participants approached? *E.g. face-to-face, telephone, mail, email* | 4 |
| 12. Sample size | How many participants were in the study? | 6 |
| 13. Non-participation | How many people refused to participate or dropped out? Reasons? | 6 |
| Setting | | |
| 14. Setting of data collection | Where was the data collected? *E.g. home, clinic, workplace* | 5 |
| 15. Presence of non-participants | Was anyone else present besides the participants and researchers? | 4 |
| 16. Description of sample | What are the important characteristics of the sample? *E.g. demographic data, date* | 7 |
| Data collection | | |
| 17. Interview guide | Were questions, prompts, guides provided by the authors? Was it pilot tested? | 4 |
| 18. Repeat interviews | Were repeat interviews carried out? If yes, how many? | 4, 6 |
| 19. Audio/visual recording | Did the research use audio or visual recording to collect the data? | 6 |
| 20. Field notes | Were field notes made during and/or after the interview or focus group? | 6 |
| 21. Duration | What was the duration of the interviews or focus group? | 6 |
| 22. Data saturation | Was data saturation discussed? | 8 |
| 23. Transcripts returned | Were transcripts returned to participants for comment and/or correction? | 6 |
| Domain 3: analysis and findings | | |
| Data analysis | | |
| 24. Number of data coders | How many data coders coded the data? | 6 |
| 25. Description of the coding tree | Did authors provide a description of the coding tree? | 8 |
| 26. Derivation of themes | Were themes identified in advance or derived from the data? | 6 |
| 27. Software | What software, if applicable, was used to manage the data? | 6 |
| 28. Participant checking | Did participants provide feedback on the findings? | 6 |
| Reporting | | |
| 29. Quotations presented | Were participant quotations presented to illustrate the themes/findings? Was each quotation identified? *E.g. participant number* | 7-17 |
| 30. Data and findings consistent | Was there consistency between the data presented and the findings? | 7-17 |
| 31. Clarity of major themes | Were major themes clearly presented in the findings? | 7-17 |
| 32. Clarity of minor themes | Is there a description of diverse cases of discussion of minor themes? | 7-17 |
